# KLF4 alleviates cerebral vascular injury by ameliorating vascular endothelial inflammation and regulating tight junction protein expression following ischemic stroke

**DOI:** 10.1186/s12974-020-01780-x

**Published:** 2020-04-07

**Authors:** Xinyu Zhang, Lu Wang, Zhenxiang Han, Jing Dong, Defang Pang, Yuan Fu, Longxuan Li

**Affiliations:** 1 Department of Neurology, Gongli Hospital, The Second Military Medical University, 219 Miaopu Road, Pudong New Area, Shanghai, 200135 People’s Republic of China; 2 The Graduate School, Ningxia Medical University, Yinchuan, Ningxia 750004 People’s Republic of China; 3 Department of Neurology and Rehabilitation, Seventh People’s Hospital of Shanghai University of TCM, Shanghai, 200137 People’s Republic of China; 4 Department of Pharmacy, Gongli Hospital, The Second Military Medical University, Shanghai, 200135 People’s Republic of China; 5 Department of Special Outpatient Service, Gongli Hospital, The Second Military Medical University, Shanghai, 200135 People’s Republic of China; 6 Department of Neurology, The Fourth Affiliated Hospital, Harbin Medical University, Harbin, 150001 People’s Republic of China

**Keywords:** Cerebral ischemic stroke, Blood-brain barrier, Cell adhesion molecules, Kruppel-like transcription factor 4

## Abstract

**Background:**

Although inflammatory cell adhesion molecules (CAMs) and anti-inflammation factor Kruppel-like transcription factor (KLF) 4 have all been reported to be induced after cerebral ischemic stroke (CIS), the close temporal and spatial relationship between expressions of CAMs and KLF4 following CIS and whether and how CAMs and KLF-4 contribute to the development of CIS-induced vascular injury are still unclear.

**Methods:**

Here, we first examined the correlation between serum levels of CAMs/KLF4 and infarct volume in acute CIS patients. Then, we determined the relationship between CAMs and KLF4 in mice after focal cerebral ischemia. Finally, we investigated the mechanism of KLF4 in protecting against oxygen-glucose deprivation-induced brain endothelial cell injury.

**Results:**

Our results demonstrated that patients with moderate to severe CIS had higher serum levels of three CAMs including E-selectin, inter-cellular adhesion molecule 1 (ICAM-1), and vascular cell adhesion molecule 1 (VCAM-1) but lower levels of KLF4 at 48 h after an acute event as compared to patients with minor CIS. The expression levels of three CAMs as well as KLF4 all correlated well with the infarct volume in all the CIS subjects at that time. Although the expressions of three CAMs and KLF4 were all induced in the ischemic hemisphere following focal cerebral ischemia, the peak timing and distribution patterns of their expression were different: the induction of KLF4 lagged behind that of the CAMs in the ischemic penumbra; furthermore, the dual immunofluorescent studies displayed that high expression of KLF4 was always associated with relatively less cerebral vascular endothelial inflammation response in the ischemic hemisphere and vice versa. Mechanistic analyses revealed that KLF4 alleviated CIS-induced cerebral vascular injury by regulating endothelial expressions of CAMs, nuclear factor-kB, and tight junction proteins.

**Conclusions:**

These data indicate that KLF4 confers vascular protection against cerebral ischemic injury, suggesting that circulating CAMs and KLF4 might be used as potential biomarkers for predicting the prognosis of acute ischemic stroke and also providing a new proof of concept and potential targets for future prevention and treatment of CIS.

## Introduction

Acute cerebral ischemic stroke (CIS) is caused by the sudden occlusion of a cerebral artery, leading to the progressive infarction of the brain and subsequent neurological deficits [1].

Early after the onset of CIS, endothelial cells of the arterial wall lose their tight junction and express inflammatory cell adhesion molecules (CAMs) such as E-selectin, inter-cellular adhesion molecule 1 (ICAM-1), and vascular cell adhesion molecule 1 (VCAM-1) [2–4]. These CAMs are widely believed to play an important role in leukocyte adherence to endothelium and leukocyte transmigration to inflammation sites [2, 5]. They contribute to the inflammatory response after ischemic brain injury. Consequently, the alteration in endothelial adhesive proteins affects not only vascular permeability but also the vascular responses to the changes in the perivascular environment [6], which may deteriorate the blood-brain barrier (BBB) injury and worsen the brain tissue damage. The long-lasting BBB disruption can directly contribute to cerebral edema and the influx of immune cell and inflammatory materials, ultimately resulting in the neuronal death, damage to the brain tissue, and neurological deficits, which plays a dominant role in the pathophysiological process of cerebral ischemic injury [7, 8].

However, immediately after the ischemic insult, many spontaneous protective mechanisms are activated to maintain cell homeostasis [9]. The Kruppel-like transcription factor 4 (KLF-4) is an evolutionarily conserved zinc finger-containing transcription factor involved in a variety of cellular functions by activating or repressing the transcriptional activity of multiple genes [10]. KLF4 is shown to repress arterial inflammation to regulate neointimal formation following vascular injury by inhibiting tumor necrosis factor-α-induced expression of VCAM- 1[11]. KLF4 is also found to be upregulated in the brain following ischemic injury [12, 13]. Importantly, KLF4 can protect brain microvascular endothelial cells from ischemic stroke-induced apoptosis [12].

There is also evidence that KLF4 is required for the maintenance of endothelial and vascular integrity in the adult animal [14]. Moreover, KLF4 regulates blood-tumor barrier permeability by altering the expressions of tight junction-related proteins [15].

Although the CAMs as well as KLF4 have all been reported to be induced after CIS, the temporal and spatial expression patterns of these molecules and cell types of their expression have yet to be fully addressed. In addition, the intricate relationship between inflammatory CAMs, KLF4, and vascular integrity after CIS is relatively unexplored and whether and how CAMs and KLF4 contribute to the development of CIS-induced vascular injury are still unclear. In light of these unanswered questions, the aim of the current study was to investigate the temporal and spatial relationship between changes of CAMs and KLF4 and BBB dysfunction and uncover the molecular mechanisms of KLF4 in protecting against vascular injury following CIS.

## Materials and methods

### Patients

Seventy-four patients with first-ever anterior circulation acute CIS were recruited from January 1, 2019, to October 31, 2019, at the Gongli Hospital, Shanghai, China. The included patients had to be admitted within 48 h from the onset of stroke or transient ischemic attack (TIA). The diagnosis had to be corroborated by a neurologist’s investigation and cerebral imaging (magnetic resonance imaging (MRI) or a computed tomography scan). Exclusion criteria were intracranial hemorrhage, pregnancy, presenting with a stroke with an undetermined time of onset, cancer, hematological diseases, severe renal or liver failure, recent myocardial infarction (less than 3 months previously), and ongoing treatment with anti-inflammatory drugs. Stroke severity was assessed using the National Institutes of Health Stroke Scale (NIHSS). The minor stroke was defined as NIHSS score < 7 and moderate to severe stroke defined as NIHSS score ≥ 7 [16]. Thirty-three age- and sex-matched healthy individuals from the Gongli Hospital were selected as the controls.

The study was performed in accordance with the principles of the Helsinki Declaration and was approved by the Ethics Committee of Gongli Hospital, Pudong New Area, Shanghai and all study participants signed a consent form before enrollment.

### Evaluation of cerebral infarct volume

Diffusion-weighted MRI (DWI) images were used to calculate infarct sizes of the patients (3.0 Tesla, Toshiba Vantage Titan) [17]. The regions of interest were defined and the infarct volume was calculated manually. The MRI was performed 48 h (accepted range 48 ± 2 h) after symptom onset. Imaging analyses were performed by trained readers blinded to all clinical and laboratory data.

### Blood sample collection and inflammatory cell adhesion molecules and KLF4 measurement

The blood samples were drawn from the forearms of acute CIS patients at 48 h after onset of the acute CIS event. The sera were immediately separated by centrifugation, and all specimens were immediately aliquoted, frozen, and stored in a dedicated − 80 °C freezer for further analysis.

The serum concentrations of three CAMs (E-selectin, VCAM-1, and ICAM-1) and KLF4 were determined by enzyme-linked immunosorbent assay (ELISA; Cusabio, Wuhan, China) according to the manufacturer’s protocol. The laboratory technician was blinded to patients’ identity.

### Experimental animals

Male C57Bl/6 mice weighing 20–25 g at the time of surgery were used for all experiments. The present study was conducted in accordance with NIH guidelines for the care and use of animals in research and under protocols approved by the Animal Care and Use Committee of Gongli Hospital, Pudong New Area, Shanghai.

### MCAO model

Focal cerebral ischemia was induced by reversible right middle cerebral artery occlusion (MCAO) surgery under pentobarbital anesthesia, followed by reperfusion as described previously [18]. A laser-Doppler perfusion monitor (LDPM, PeriFlux5000, Perimed, Sweden) was used for the measurement of cerebral blood flow (CBF). The CBF was controlled by adjusting the filament in the artery for the induction of ischemia. Only the mice whose CBF showed a drop of over 85% of baseline (before MCAO) just after MCAO were included for further experiment [19]. At the end of ischemia (90 min MCAO), mice were briefly re-anesthetized, and reperfusion was initiated by filament withdrawal. Sham animals (control) were subjected to the same procedure but did not receive MCAO. Mice were euthanized 0, 1, 2, 4, 7, 14 days post-ischemia.

### Immunohistochemistry studies and antibodies

Immunofluorescent (IF) staining was performed to examine how cerebral ischemia influences the expression of three CAMs (E-selectin, VCAM-1, and ICAM-1) and KLF4. Mice at different time points of reperfusion were euthanized by perfusion with ice-cold saline, and the brains were rapidly dissected and stored at − 80 °C. IF studies were performed as previously described on 10-μm-thick frozen coronal sections [18]. The following monoclonal antibodies from BD Pharmingen (La Jolla, CA) were used in this study: FITC conjugated rat anti-mouse CD31 (PECAM-1) (clone MEC13.3, 553372, 1:200) and FITC-conjugated rat anti-mouse Mac-1 (CD11b) (clone M1/70, 553310, 1:100). The mouse anti-E-selectin monoclonal antibody (SC-137054, 1:100), mouse anti-ICAM-1 monoclonal antibody (SC-8439, 1:100), and mouse anti-VCAM-1 monoclonal antibody (SC-13160, 1:100) were obtained from Santa Cruz Biotechnology, Inc. The rabbit anti-KLF4 polyclonal antibody (ab129473, 1:500) and Armenian hamster anti-CD31 monoclonal antibody (2H8, ab119341, 1:250) were obtained from Abcam. The Cy3-conjugated mouse anti-glial fibrillary acidic protein (GFAP) (clone G-A-5, 1:1500) was purchased from Sigma (St. Louis, MO, USA). The rat anti-ZO-1 monoclonal antibody (R40.76, MABT11, 1:100, Merck Millipore, Darmstadt, Germany), mouse anti-Claudin 5 monoclonal antibody (4C3C2, 35-2500, 1:200, Invitrogen, Camarillo, CA, USA), and Alexa Fluor 488-conjugated goat anti-rat secondary antibody were obtained from Invitrogen (Carlsbad, CA, USA). Alexa Fluor 488-conjugated goat anti-rabbit, Cy3-conjugated goat anti-rabbit, anti-Armenian hamster, and anti-mouse secondary antibody were obtained from Jackson Immunoresearch (West Grove, PA, USA); Alexa Fluor 488-conjugated goat anti-mouse secondary antibody was obtained from Bioss. The negative controls for staining and confocal imaging were used to confirm a coexistence of the vessel proteins.

Quantification of the number of positive cells for the different antigens was performed as previously reported [20, 21]. In brief, images of the region of interest were acquired using a × 20 objective on a Leica TCS SP5 II microscope to determine the number of positive events per field of view (FOV). A minimum of three serial brain sections per mouse was selected for analysis of each antigen and matched between mice so that the approximate position of sections used for IF staining was equivalent between different experimental conditions. Three images were taken from the ischemic penumbra including cortex and striatum as well as ischemic core of each brain section and quantified by eye for the number of positive events per FOV. The number of antigen-positive events per FOV for each section was calculated as the mean of total numbers obtained from the three regions. These averages of three brain sections were used for statistical analysis for each mouse.

### Cell culture

Immortalized mouse brain endothelial cells (BECs) of the bEnd3 cell line were obtained from Shanghai Bioleaf Biotech Co., Ltd. Cells were grown on six-well plates pre-coated with type I or IV collagen (10 μg/ml, Sigma, for 2 h at 37 °C) cultured in endothelial basal medium (EBM-2) (Lonza, CC-3156) supplemented with 10% FBS (Gibco), ascorbic acid, L-glutamine, penicillin/streptomycin, and human basic fibroblast growth factor (bFGF) (all from Sigma). Cells were maintained in a humidified incubator at 37 °C and 5% CO_2_, and the medium was changed every 48 h.

### Construction and transfection of siRNA and pcDNA3.1 plasmids

Small interfering RNA (siRNA) and plasmid construction were performed as previously reported [22, 23]. Briefly, three specific sequences of siRNA targeting the murine KLF4 (①sense 5′-GGUUUAUAUUGAAUCCAAAGA-3′, antisense 5′-UUUGGAUUCAAUAUAAACCGG-3′; ② sense 5′-GGUCAUCAGUGUUAGCAAAGG-3′, antisense 5′-UUUGCUAACACUGAUGACCGA-3′; and ③ sense 5′-GGAGAAAGGAAGAGUUCAAGA-3′, antisense 5′-UUGAACUCUUCCUUUCUCCUG-3′) were designed and synthesized by Gene Pharma (Shanghai, China). Murine KLF4 (NM_010637.3) coding sequence was cloned into pCDNA3.1 plasmid vector (Invitrogen, Carlsbad, CA, USA) through EcoRI and XhoI sites. Following the manufacturer’s instructions, confluent bEnd3 cells were transfected with siRNAs or sequencing-verified constructs using Lipofectamine 3000 (Invitrogen). After 48 h of transfection, bEnd3 cells were harvested for the analysis of function and gene expression. The control siRNA treated or mock-transfected bEnd3 cells were used as negative control.

### Oxygen–glucose deprivation and restoration (OGD/R)

Forty-eight hours after transfection, bEnd3 cell cultures were subjected to ischemia-like injury through oxygen-glucose deprivation (OGD) for 4 h by placing cultures in an anaerobic chamber (Forma, Thermo Scientific, Asheville, NC, USA) with an atmosphere of 5% CO_2_ and 95% N_2_ in a deoxygenated glucose-free balanced salt solution (BSS0). After 4 h of OGD, cultures were returned to control conditions (restoration) by adding 5.5 mM glucose to the media under normoxic conditions. Control cultures (no injury) were incubated with a balanced salt solution containing 5.5 mM glucose (BSS5.5). All cultures were maintained in a humidified 37 °C incubator.

### RNA extraction, reverse-transcription, and qPCR

Quantitative real-time PCR (qPCR) analysis was used to determine the mRNA expression of E-selectin, VCAM-1, ICAM-1, KLF4, and tumor necrosis factor (TNF)-α in brain tissue or cultured cells. The brain samples were taken from the ipsilateral ischemic cerebral cortex at different time points of reperfusion after MCAO and cell lysate was obtained from cultured bEnd3 cells at 12 h of restoration following OGD (*n* = 4/group). Total RNA was extracted with TRIzol reagent (Invitrogen, Carlsbad, CA) according to the manufacturer’s instructions. RNA was reverse transcribed into cDNA using a specific primer and a RevertAid First Strand cDNA Synthesis Kit (Thermo). qPCR was conducted using FastStart Universal SYBR Green Master (Rox) (Roche) and an ABI stepone-plus Real-time PCR system. Forward and reverse primer sets for each cDNA were used as follows: 5′-CTGCGAAGAAGGATTTGAACTGA-3′ and 5′-CTTGGACATTGTACCACTTGGC-3′ (for E-selectin, NM_011345.2 ); 5′-AGATAGACAGCCCACTAAACGC-3′ and 5′-CAGCCTGTAAACTGGGTAAATGT-3′ (for VCAM-1, NM_011693.3); 5′-CTCGGAAGGGAGCCAAGTAAC-3′ and 5′-CAGCCGAGGACCATACAGCA-3′ (for ICAM-1, NM_010493.3 ); 5′-CGACTAACCGTTGGCGTGA-3′ and 5′-TGGGTTAGCGAGTTGGAAAGG-3′ (for KLF4, NM_010637.3 ); 5′-ACCCTCACACTCACAAACCA-3′ and 5′- ATAGCAAATCGGCTGACGGT-3′ (for TNF-α, NM_001278601.1); and 5′-CCTCGTCCCGTAGACAAAATG-3′ and 5′-TGAGGTCAATGAAGGGGTCGT-3′ (for GAPDH, NM_008084.2). The average cycle threshold (Ct) value was normalized using the GAPDH signal. Relative transcript levels were calculated using the 2^-△△CT^ method. Each mRNA level was expressed as the fold-increase over the level of sham control or NO-OGD/R control group.

### Western blot analysis

Twelve hours after the restoration of OGD, bEnd3 cells were harvested and lysed with lysis buffer (1% NP-40, 50 mM Tris HCl, pH 8.0, 150 mM sodium chloride) supplemented with protease and phosphatase inhibitor cocktails. Protein concentration was determined using the BCA protein assay kit (Eppendorf-Bio photometer, Germany). Western blotting and semi-quantitative analyses were performed as described previously [18]. The following primary antibodies were purchased from Invitrogen (Carlsbad, CA, USA): Armenian hamster anti-ICAM-1 monoclonal antibody (3E2B, MA5405, 1:20), rabbit anti-nuclear factor-κB (NF-κB) polyclonal antibody (51-3500, 1:1000), rabbit anti-p-NF-κB polyclonal antibody (PA5-37658, 1:1000), rabbit anti-E-selectin monoclonal antibody (15, MA5-29785, 1:1000), rabbit anti-VCAM-1 monoclonal antibody (SA05-04, MA5-31965,1:1000), rabbit anti-KLF4 (PA5-27440,1:5000), rabbit anti-Claudin-5 polyclonal antibody (34-1600, 1:170), and rabbit anti-zonula occludens-1 (ZO-1) polyclonal antibody (61-7300,1:1000). β-actin was obtained from Neomarker (1:1000, Fremont, CA). Within each sample, levels of proteins were first normalized to the level of β-actin and then expressed as the fold-increase over the level of NO-OGD/R control group.

### Statistical analysis

Categorical variables were expressed as counts (percentage) and continuous variables as mean ± standard deviation unless otherwise indicated. Statistical significance was assessed by the *t* test, chi-square test, and one- or two-way analysis of variance (ANOVA), and a Bonferroni post hoc test was used to test multiple comparisons. Correlations were assessed using Pearson’s method, with log transformation of non-normally distributed data prior to statistical analysis. All statistical analyses were performed with SPSS (version16.0; SPSS, Chicago, IL, USA) and significance was defined as *P* < 0.05.

## Results

### Demographic and clinical characteristics

Seventy-four patients with CIS and thirty-three healthy controls were included in this study, of whom 39 presented with minor strokes (NIHSS score < 7) and 35 with moderate to severe stroke (NIHSS score ≥ 7). The patients and the controls’ clinical characteristics are presented in Table 1.

**Table 1:** Baseline clinical characteristics of acute stroke patients and healthy controls

Characteristics	Controls (*n* = 33)	NIHSS < 7 (*n* = 39)	NIHSS ≥ 7 (*n* = 35)
Age mean ± SD (years)	64.92 ± 9.16	65.41 ± 10.03	67.54 ± 12.57
Male, *n* (%)	19 (57.58%)	25 (64.1%)	24 (68.57)
Hypercholesterolemia, *n* (%)	9 (27.27%)	20 (51.28%)*	19 (54.29%)*
Hypertension, *n* (%)	15 (45.45%)	26 (66.67%)	26 (74.29%)*
Diabetes, *n* (%)	4 (12.12%)	11 (28.21%)	12 (34.29%)
Active smoker, *n* (%)	8 (24.24%)	10 (25.64%)	15 (42.86%)
Alcohol, *n* (%)	5 (15.15%)	8 (20.51%)	13 (37.14%)
Atrial fibrillation, *n* (%)	3 (9.09%)	6 (15.38%)	11 (31.43%)*
Coronary disease, *n* (%)	–	3 (7.69%)	5 (14.26%)
Previous antiplatelet treatment, *n* (%)	10 (30.3%)	12(30.77%)	13(37.14%)
Previous treatment with statin, *n* (%)	9 (27.27%)	14 (35.9%)	15 (42.86%)

Minor stroke was defined as NIHSS score < 7, moderate to severe stroke defined as NIHSS score ≥ 7

*NIHSS* National Institute of Health Stroke Scale, *SD* standard deviation

*P < 0.05 vs healthy controls

The frequency rate of hypercholesterolemia in the minor stroke patients and the frequency rates of hypercholesterolemia, hypertension, and atrial fibrillation in the moderate to severe stroke patients were significantly higher than that of the controls (all *P* < 0.05). However, no significant difference was observed between the minor and moderate to severe stroke groups in terms of age, gender, hypercholesterolemia, hypertension, diabetes, nicotine/alcohol use, atrial fibrillation and coronary disease, and with regards to antiplatelet or statin treatments before stroke (Table 1).

### Patients with moderate to severe stroke have higher serum levels of three cell adhesion molecules but lower levels of KLF4 at 48 h after ischemic onset as compared to patients with minor stroke

The serum levels of three CAMs (E-selectin, VCAM-1, and ICAM-1) and KLF4 in the minor and moderate to severe stroke patients and the controls are shown in Table 2. Compared to the controls, patients in minor stroke group and moderate to severe stroke group showed significantly increased serum levels of E-selectin, VCAM-1, and ICAM-1 at 48 h after ischemic stroke onset (minor stroke vs controls: all *P* < 0.05 for E-selectin, VCAM-1, and ICAM-1; moderate to severe stroke vs controls: all *P* < 0.001 for E-selectin, VCAM-1, and ICAM-1 ); As compared to minor stroke patients, the serum levels of E-selectin, VCAM-1 and ICAM-1 were all markedly higher at 48 hours after ischemic stroke onset in moderate to severe stroke patients (all *P* < 0.001 for E-selectin, VCAM-1, and ICAM-1). However, compared to the controls, the minor stroke patients exhibited, but moderate to severe stroke patients did not show significantly increased serum levels of KLF4 at 48 h after ischemic stroke onset (minor stroke vs controls: *P* < 0.001); As compared to minor stroke patients, the serum levels of KLF4 were significantly lower at 48 h after ischemic stroke onset in moderate to severe stroke patients (*P* < 0.01).

**Table 2:** The comparison of serum levels of three cell adhesion molecules, KLF4, and infarct volume at 48 h after ischemic onset in patients with minor and moderate to severe stroke and healthy controls

Variable	Controls (*n* = 33)	NIHSS < 7 (*n* = 39)	NIHSS ≥ 7 (*n* = 35)
E-selectin, ng/ml, mean ± SD	1.32 ± 0.91	3.02 ± 2.61*	6.28 ± 4.25***^###^
VCAM-1, ng/ml, mean ± SD	151.75 ± 58.21	227.1 ± 96.87*	343.5 ± 181.7***^###^
ICAM-1, ng/ml, mean ± SD	30.51 ± 15.83	54.68 ± 42.45*	93.99 ± 55.94***^###^
KLF4, pg/ml, mean ± SD	185.47 ± 87.93	350 ± 180.5***	217.4 ± 189.2^##^
Infarct volume, ml, mean ± SD	–	7.24 ± 8.36	33.37 ± 30.59^###^

Minor stroke was defined as NIHSS score < 7, moderate to severe stroke defined as NIHSS score ≥ 7

*ICAM-1* intercellular adhesion molecule-1, *KLF4* Kruppel-like factor 4, *NIHSS* National Institute of Health Stroke Scale, *SD* standard deviation, *VCAM-1* vascular cell adhesion molecule-1

*P < 0.05 vs healthy controls

***P < 0.001 vs healthy controls

^##^*P* < 0.01 vs NIHSS < 7 group

^###^*P* < 0.001 vs NIHSS < 7 group

As expected, the infarct volume at 48 h after stroke onset in moderate to severe stroke patients was also significantly larger than that of the minor stroke patients (*P* < 0.001).

### The infarct volume is correlated with the serum levels of three cell adhesion molecules and KLF4 at 48 h after ischemic onset in the acute ischemic stroke patients

**Fig. 1:**
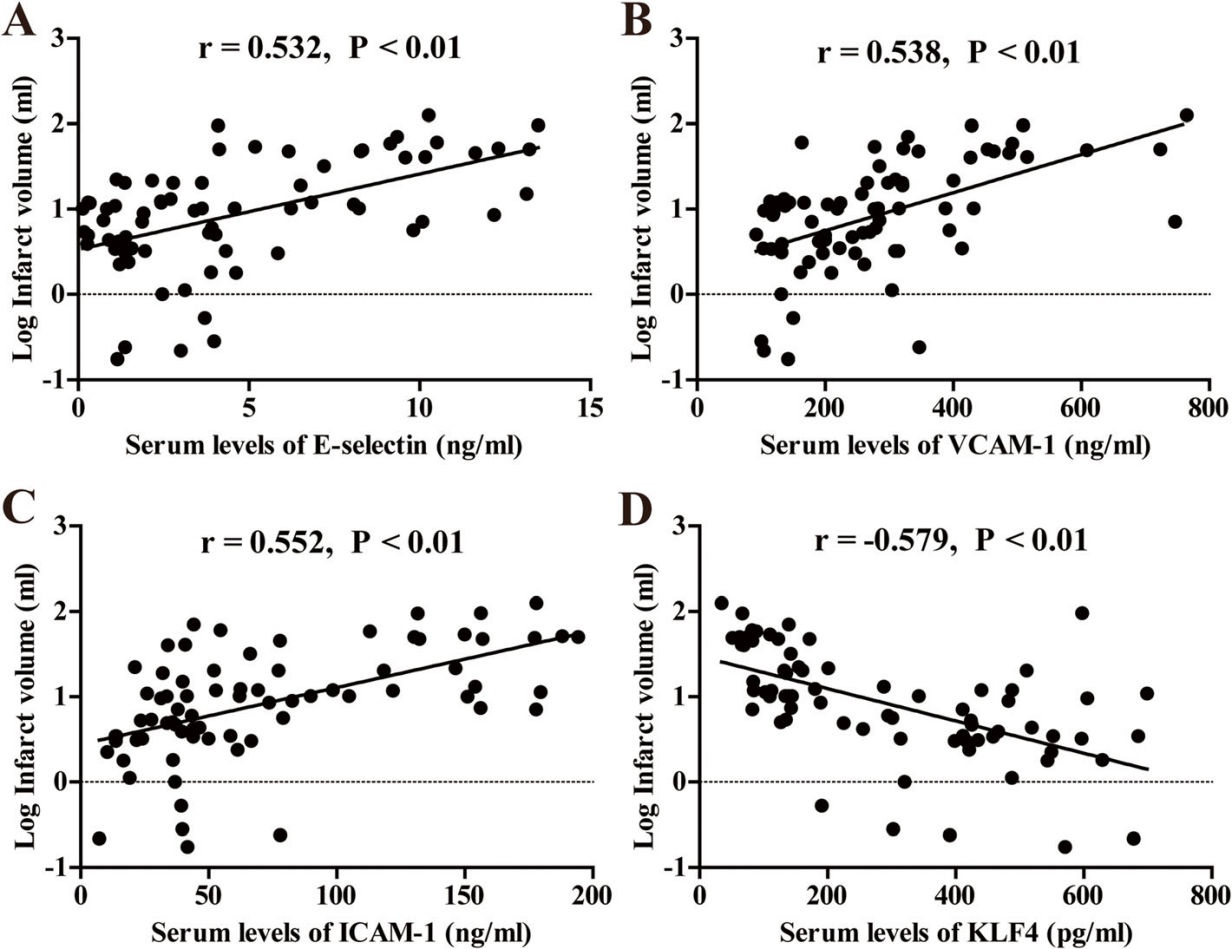
The correlations of infarct volume with the serum levels of three cell adhesion molecules and KLF4 at 48 h after ischemic onset in the acute ischemic stroke patients. Serum levels of three cell adhesion molecules (E-selectin, VCAM-1, and ICAM-1) and KLF4 at 48 h after ischemic onset were measured using ELISA. Diffusion-weighted MRI images were used to calculate the infarct volume. After log transformation, the Pearson analyses revealed that the log infarct volume was significantly positively correlated with serum levels of three cell adhesion molecules (E-selectin, VCAM-1, and ICAM-1) and markedly negatively correlated the serum levels of KLF4 in all the subjects (*n* = 74)

The Pearson analyses revealed that the infarct volume was positively correlated with the serum levels of all the three CAMs (E-selectin, VCAM-1, and ICAM-1), but negatively correlated with the serum levels of KLF4 at 48 h after ischemic stroke onset in all the CIS subjects (*r* = 0.532, *P* < 0.01 for infarct volume with E-selectin; *r* = 0.538, *P* < 0.01 for infarct volume with VCAM-1; *r* = 0.552, *P* < 0.01 for infarct volume with ICAM-1; and *r* = − 0.579, *P* < 0.01 for infarct volume with KLF4) (Fig. 1).

### The expressions of three cell adhesion molecules and KLF4 upregulate in the ischemic hemisphere following focal cerebral ischemia

**Fig. 2:**
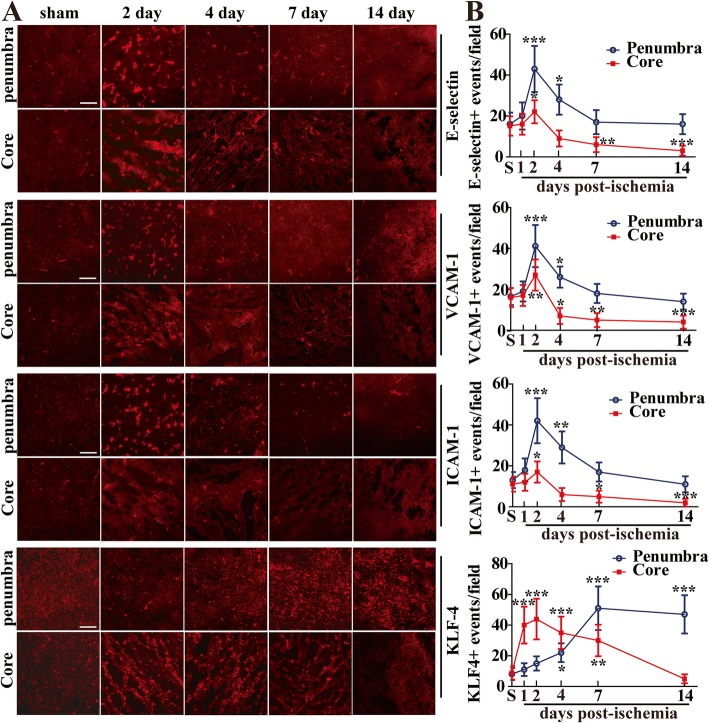
Increased expression of three cell adhesion molecules and KLF4 following focal cerebral ischemia. **a** Images show IF staining for three cell adhesion molecules (E-selectin, VCAM-1, and ICAM-1) and KLF4 in ischemic hemisphere from sham-operated mice (S, control) or mice at day 1, 2, 4, 7, and 14 post-ischemia. Scale bar = 100 μm. **b** Quantification of E-selectin, VCAM-1 and ICAM-1, and KLF4 expressions. Results are expressed as the mean ± standard deviation of the number of positive events per field of view (*n* = 6 per experimental group). Note that cerebral ischemia induced a strong increase in the expression of all the three cell adhesion molecules in both ischemic penumbra and core, reaching a peak at day 2 and then declining at day 4. While in the ischemic penumbra, the number of KLF4-positive events increased slightly during the first 2 days post-ischemia, but then increased significantly by day 4, and reached a maximum between 7 and 14 days post-ischemia. However, in the ischemic core, the number of KLF4-positive events increased markedly during the first 2 days post-ischemia, before declining at day 4. *P < 0.05, **P < 0.01, ***P < 0.001 compared with control

The IF staining showed that the numbers of three CAMs including E-selectin, VCAM-1 and ICAM-1-positive events/field increased in both ischemic penumbra and core, reaching a peak at day 2, and then drastically declining at day 4 (Fig. 2).

Compared with the control brain, the number of E-selectin-positive events/field at day 2 increased from 16.02 ± 5.62 to 42.98 ± 11.27 (*P* < 0.001) in the penumbra and from 15.11 ± 4.65 to 22.03 ± 5.63 (*P* < 0.05) in the core. In a similar manner, the number of VCAM-1-positive events/field increased from 16.5 ± 4.16 to 41.14 ± 10.29 (*P* < 0.001) in the penumbra, and from 15.97 ± 4.66 to 27 ± 7.59 (*P* < 0.01) in the core, and the number of ICAM-1-positive events/field increased from 13.06 ± 3.92 to 42.07 ± 11.02 (*P* < 0.001) in the penumbra, and from 11.09 ± 3.67 to 16.99 ± 5.14 (*P* < 0.05) in the core (Fig. 2a, b).

While in the ischemic penumbra, the number of KLF4-positive events increased slightly during the first 2 days post-ischemia, then increased significantly by day 4, and reached a maximum between 7 and 14 days post-ischemia. However, in the ischemic core, the number of KLF4-positive events increased markedly during the first 2 days post-ischemia, before declining at day 4.

Compared with control brain, the number of KLF4-positive events at day 7 increased from 8.12 ± 3.91 to 50.96 ± 14.21 (*P* < 0.001) in the penumbra, and the number of KLF4-positive events at day 2 increased from 8.97 ± 4.41 to 43.93 ± 13.23 (*P* < 0.001) in the core (Fig. 2a, b).

**Fig. 3:**
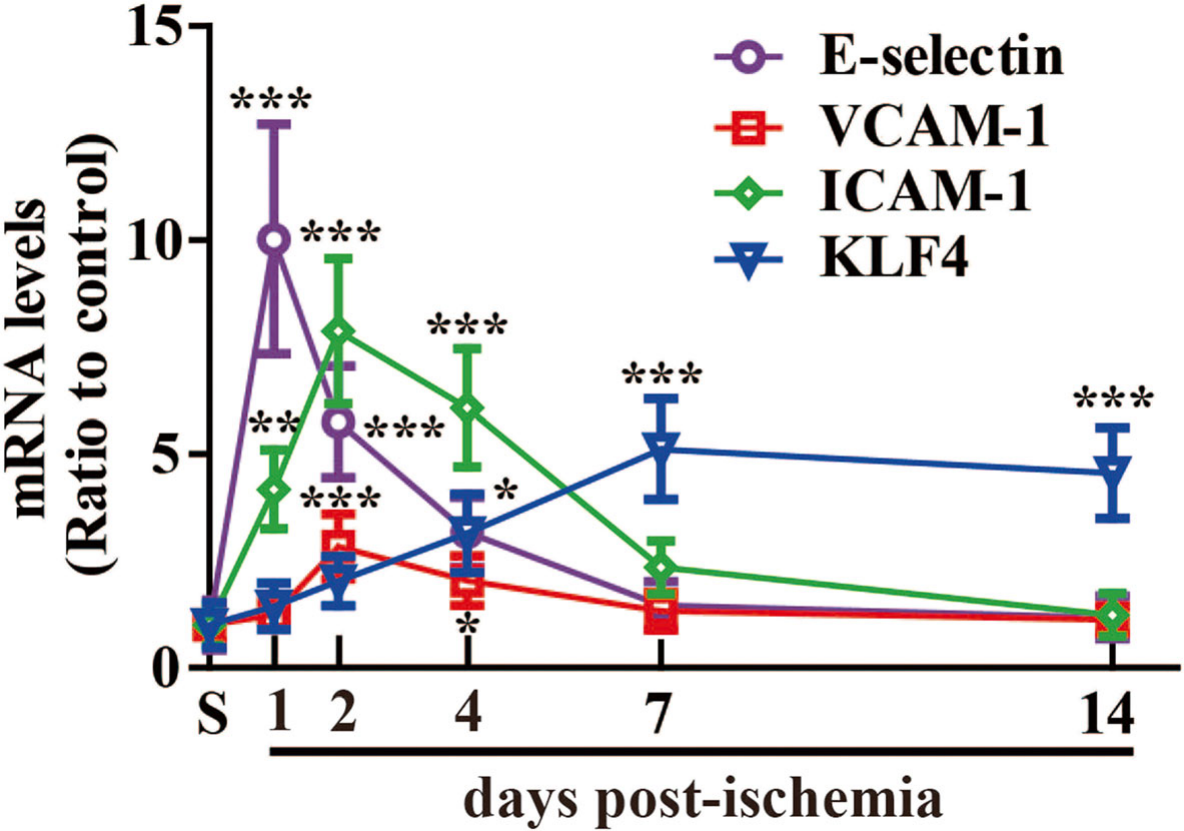
The mRNA expression of three cell adhesion molecules and KLF4 in the ischemic brain following focal cerebral ischemia. The mRNA levels of E-selectin, VCAM-1, ICAM-1, and KLF4 in the ipsilateral ischemic cerebral cortex from sham-operated mice (S, control) or mice at days 1, 2, 4, 7, and 14 post-ischemia was measured by qPCR. Results are expressed as the mean ± standard deviation (*n* = 4 per experimental group). Note that cerebral ischemia markedly increased the mRNA expression of all the three cell adhesion molecules including E-selectin, VCAM-1, and ICAM-1 in the ischemic hemisphere, reaching a peak at day 1 or 2 and then declining at day 2 or 4, while cerebral ischemia also induced a strong increase in mRNA expression of KLF4 over the entire 14-day time course, with this effect maximal between 7 and 14 days post-ischemia. *P < 0.05, **P < 0.01, ***P < 0.001 compared with control

To validate IF results, we performed qPCR to quantify mRNA levels of E-selectin, VCAM-1, and ICAM-1 as well as KLF4 in the ipsilateral ischemic cerebral cortex. As shown in Fig. 3, cerebral ischemia induced marked upregulation of the mRNA levels of all the three CAMs including E-selectin, VCAM-1, and ICAM-1 in the ischemic hemisphere, reaching a peak at day 1 or 2 and then declining at day 2 or 4. Compared with control brain (sham), E-selectin levels increased 10.02 ± 2.68-fold (*P* < 0.001) at day 1 post-ischemia, and VCAM-1 and ICAM-1 increased 2.84 ± 0.76-fold (*P* < 0.001) and 7.87 ± 1.7-fold (*P* < 0.001), respectively, at day 2 post-ischemia, while cerebral ischemia also increase the mRNA levels of KLF4 over the entire 14-day time course, with this effect maximal between 7 and 14 days post-ischemia. Compared with control brain, mRNA levels of KLF4 at day 7 increased 5.12 ± 1.18-fold (*P* < 0.001).

**Fig. 4:**
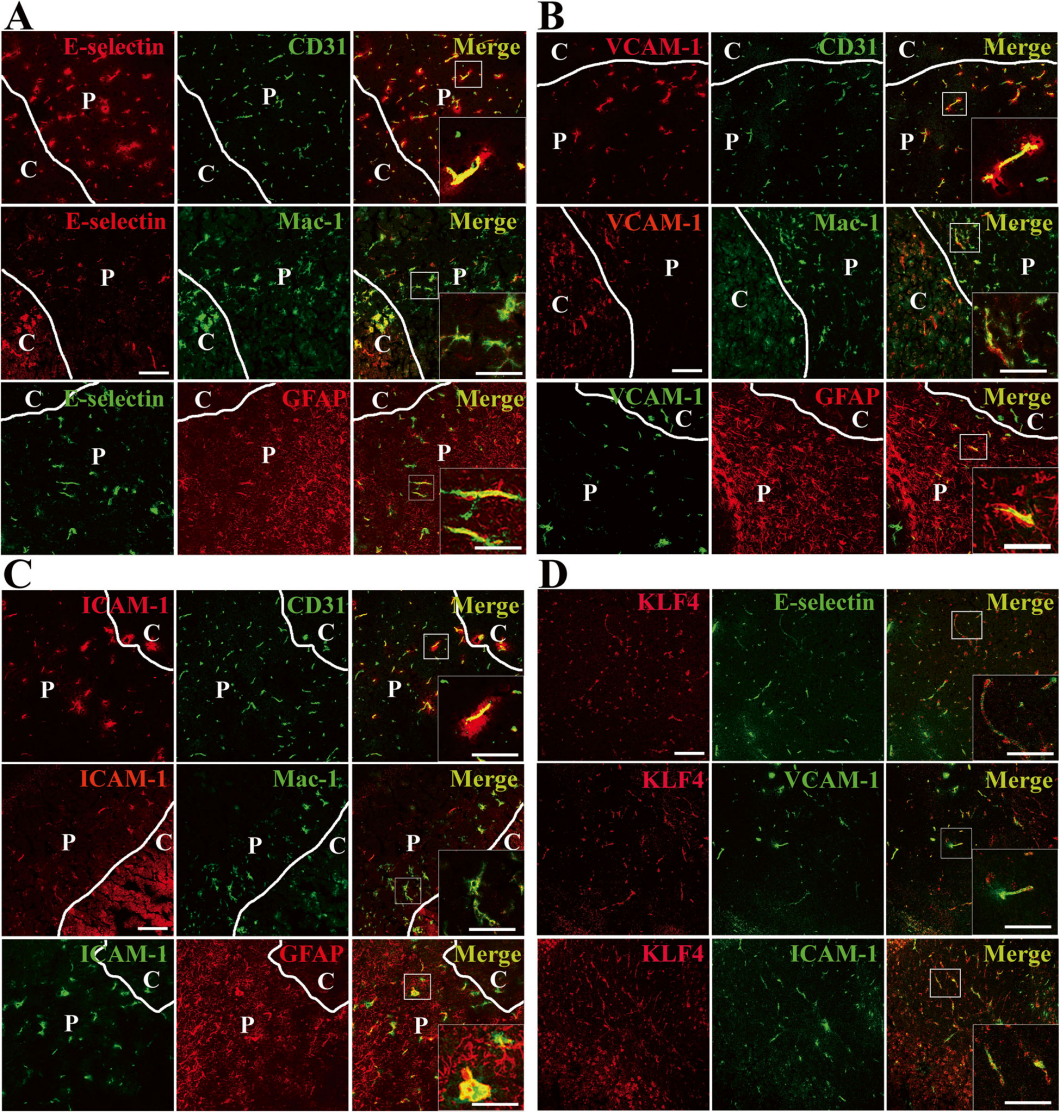
Cellular localization of three cell adhesion molecules (E-selectin, VCAM-1, and ICAM-1) expression and co-expression of E-selectin, VCAM-1, and ICAM-1 with KLF4 on cerebral blood vessels after focal cerebral ischemia. Images show the dual-IF staining for E-selectin/VCAM-1/ICAM-1 with CD31, Mac-1, GFAP, or KLF4 in ischemic hemisphere at day 2 post-ischemia. Scale bar = 100 μm (inserts = 40 μm). Note that E-selectin/VCAM-1/ICAM-1 always co-localized with CD31-positive vessels, especially on the leaked blood vessels. In addition, E-selectin/VCAM-1/ICAM-1 co-localized with quite a few Mac-1-positive microglia/inflammatory macrophages in the ischemic hemisphere. Furthermore, E-selectin/VCAM-1/ICAM-1 also co-localized with GFAP-positive astrocytes in the penumbra (penumbra = P), but in the ischemic core (core = C), this was markedly reduced. Of interest, a fraction of E-selectin/VCAM-1/ICAM-1 co-localized with KLF4, but the distribution patterns of their expressions were different, where the high levels of KLF4 were expressed, relatively low levels of E-selectin/VCAM-1/ICAM-1 were expressed on the blood vessels in the ischemic hemisphere, and vice versa.

To determine which cell type contributed to the upregulations of three CAMs (E-selectin, VCAM-1, and ICAM-1) following CIS, dual-IF was performed on frozen sections of ischemic hemisphere taken from 2 days post-ischemia, using antibodies specific for E-selectin/VCAM-1/ICAM-1(Cy-3), the endothelial-specific marker CD31 (AlexaFluor-488), and microglial markerMac-1 (AlexaFluor-488) or E-selectin/VCAM-1/ICAM-1(AlexaFluor-488), and astrocyte marker GFAP (Cy-3). As shown in Fig. 4 a–c, E-selectin/VCAM-1/ICAM-1 always co-localized with CD31-positive vessels, especially on the leaked blood vessels. In addition to vascular expression, we also noticed that E-selectin/VCAM-1/ICAM-1 co-localized with quite a few Mac-1-positive microglia/inflammatory macrophages in the ischemic hemisphere. Furthermore, E-selectin/VCAM-1/ICAM-1 also co-localized with GFAP-positive astrocytes in the penumbra, but this co-localization was markedly disrupted in the ischemic core.

**Fig. 5:**
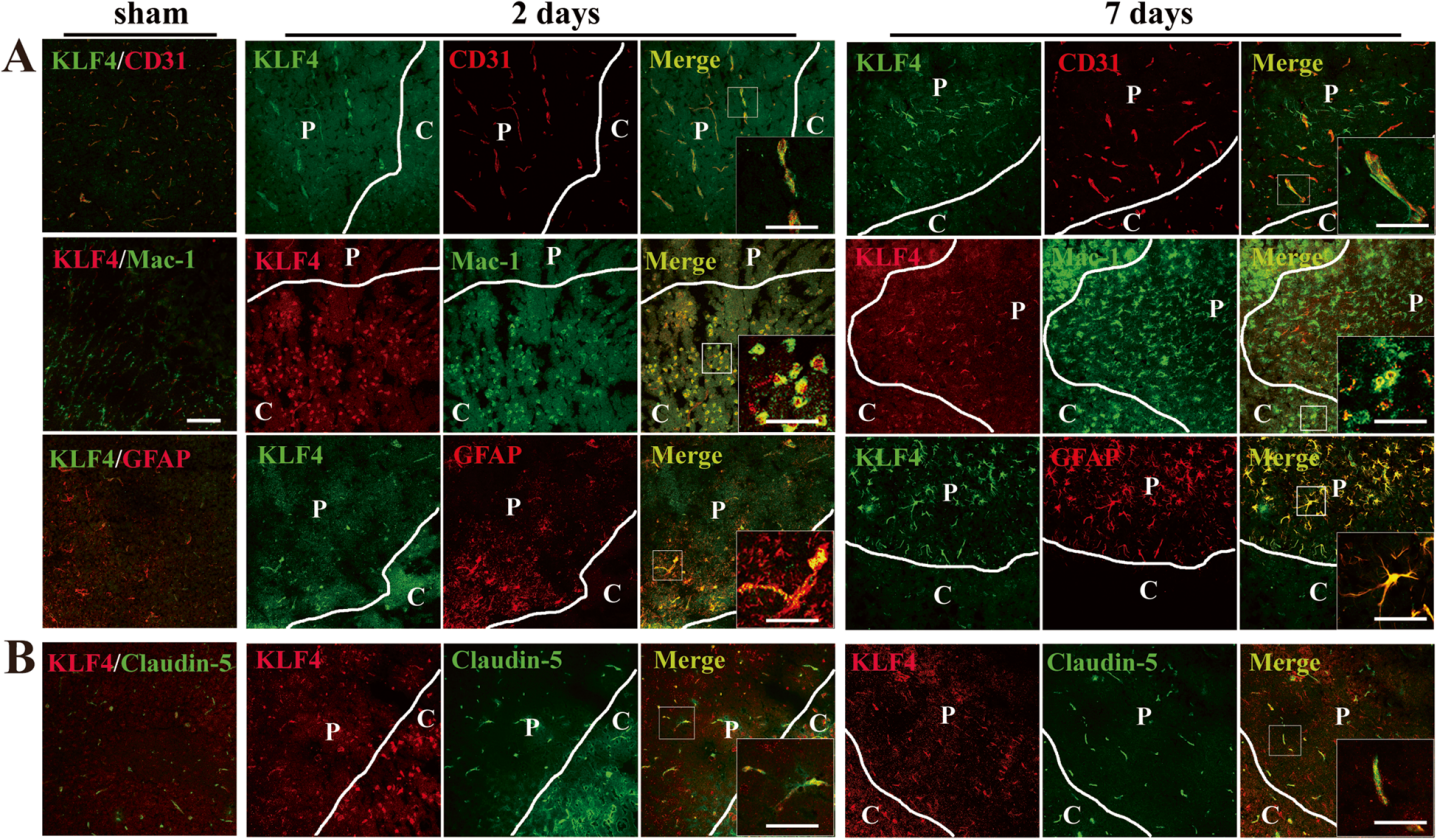
Cellular localization of KLF4 expression and co-expression of KLF4 with Claudin-5 on cerebral blood vessels after focal cerebral ischemia. Images show the dual-IF staining for KLF4 with CD31, GFAP, Mac-1, or Claudin-5 in the ischemic hemisphere from sham-operated mice or mice at days 2 and 7 post-ischemia. Scale bar = 100 μm (inserts = 40 μm). Note that KLF4 immunoreactivity was observed in CD31-positive vessels in the ischemic hemisphere at days 2 and 7 post-ischemia. At day 2 post-ischemia, KLF4 co-localized extensively with Mac-1-positive microglia/inflammatory macrophages in the ischemic hemisphere, especially in the core (core = C); at day 7, the co-expression of KLF4 with activated microglia/inflammatory macrophages can still be seen in the ischemic hemisphere, including the penumbra (penumbra = P) and the core (core = C). Furthermore, KLF4 expression always strongly co-localized with cells expressing high levels of GFAP in the ischemic penumbra (penumbra = P) at days 2 and 7 post-ischemia, especially at day 7, but this co-localization was significantly reduced in the ischemic core (core = C) (**a**). Meanwhile, KLF4 expression co-localized with Claudin-5 on the blood vessels in the ischemic hemisphere at days 2 and 7 post-ischemia (**b**)

To define the phenotype of the cells expressing KLF4 after cerebral ischemic stroke, dual-IF was performed on frozen sections of ischemic hemisphere taken from sham-operated mice (sham) or 2 and 7 days post-ischemia, using antibodies specific for KLF4 (AlexaFluor-488), the endothelial-specific marker CD31 (Cy-3) and astrocyte marker GFAP (Cy-3), or KLF4 (Cy-3) and microglial marker Mac-1 (AlexaFluor-488). As shown in Fig. 5, KLF4 immunoreactivity was observed in CD31-positive vessels in the ischemic hemisphere at days 2 and 7 post-ischemia. At day 2 post-ischemia, KLF4 co-localized extensively with Mac-1-positive microglia/inflammatory macrophages in the ischemic hemisphere, especially in the core; at day 7, the co-expression of KLF4 with activated microglia/inflammatory macrophages can still be seen in the ischemic hemisphere, including the penumbra and the core. Furthermore, KLF4 expression always strongly co-localized with cells expressing high levels of GFAP in the ischemic penumbra at days 2 and 7 post-ischemia, especially at day 7, but this co-localization was markedly disrupted in the ischemic core.

### KLF4 regulates cerebral vascular endothelial expression of cell adhesion molecules, NF-κB, and tight junction proteins following cerebral ischemia

A previous study showed that injury-induced expression of CAMs such as E-selectin and VCAM-1 in the carotid arteries was enhanced in KLF4-conditional knockout mice, indicating that KLF4 can repress arterial inflammation following vascular injury [11]. In the current study, we first performed dual-IF to examine whether E-selectin/VCAM-1/ICAM-1 (AlexaFluor-488) and KLF4 (Cy-3) show any overlap in their expression profiles on cerebral blood vessels after CIS. Of interest, a fraction of E-selectin/VCAM-1/ICAM-1 expression co-localized with KLF4 in the ischemic hemisphere at days 2 post-ischemia, but the distribution patterns of their expressions were different, where the high levels of KLF4 were expressed, relatively low levels of E-selectin/VCAM-1/ICAM-1 were expressed on the cerebral blood vessels in the ischemic hemisphere, and vice versa (Fig. 4d). It seemed that the enhanced KLF4 could suppress the vascular endothelial expression of three CAMs including E-selectin, VCAM-1, and ICAM-1 after cerebral ischemia.

**Fig. 6:**
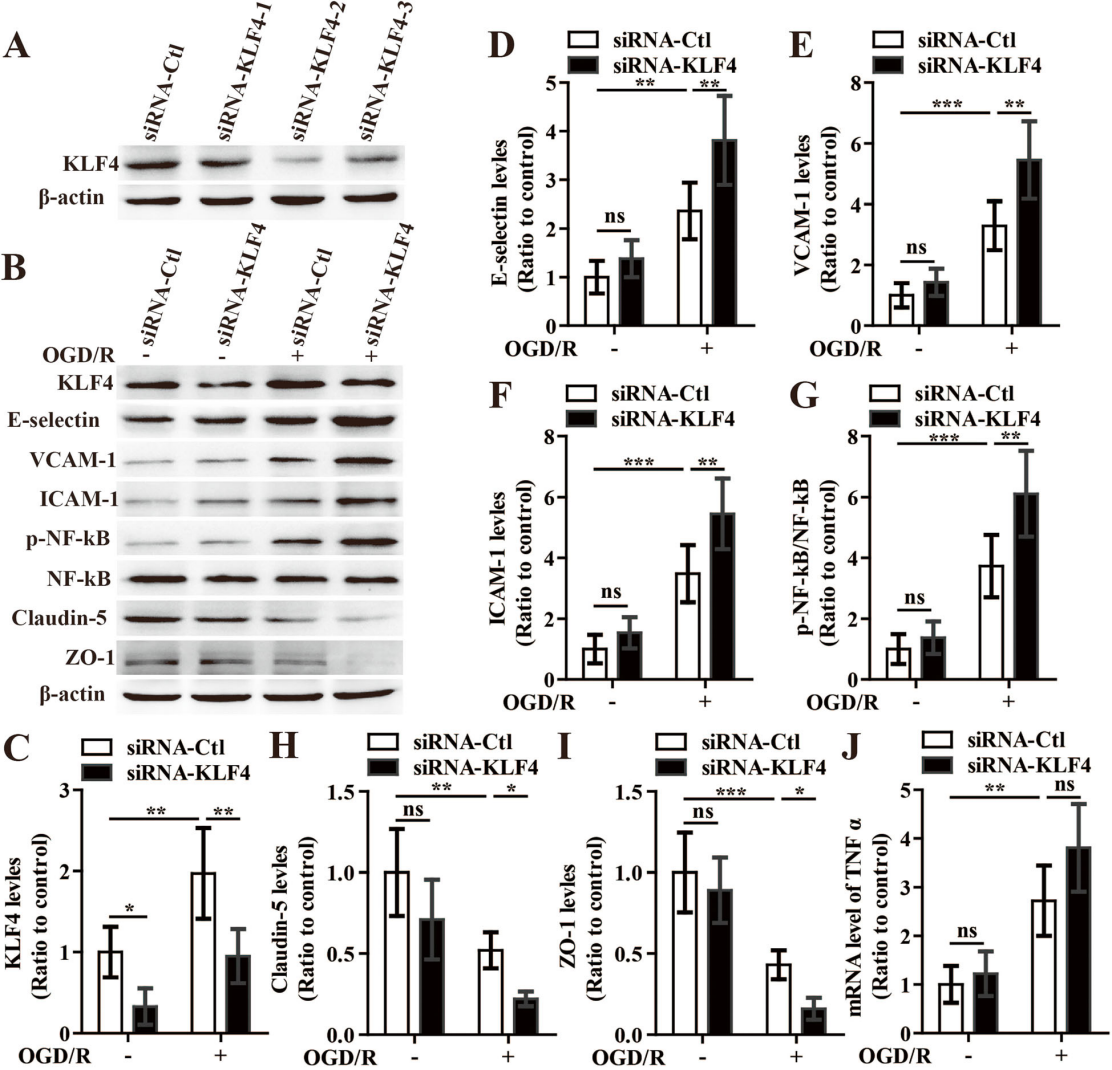
The influence of knockdown of KLF4 on the expression of three cell adhesion molecules, phosphorylated NF-κB, tight junction proteins, and TNF-α in bEnd3 cells under OGD/R conditions. **a** Representative images of western blot for the expression of KLF4 in the bEnd3 cells in the negative control siRNA (siRNA-Ctl) or three different KLF4-specific siRNA (siRNA-KLF4)-treated group. Note that three different specific siRNA against the murine KLF4 all reduced the expression of KLF4 in bEnd3 cells compared to that of siRNA-Ctl-treated bEnd3 cells, respectively, and siRNA-KLF4-2 can target the KLF4 with the highest efficiency. **b** Representative images of western blot for the expression of KLF4, E-selectin, VCAM-1, ICAM-1, p-NF-κB, Claudin-5, and ZO-1 in the bEnd3 cells of siRNA-Ctl and siRNA-KLF4-2 group at 12 h restoration of OGD or NO-OGD/R. **c**–**i** Bar graphs show the quantitative analyses of western blots as ratios of KLF4/β-actin (**c**), E-selectin/β-actin (**d**), VCAM-1/β-actin (**e**), ICAM-1/β-actin (**f**), phosphorylated NF-κB/total NF-κB (**g**), Claudin-5/β-actin (**h**), and ZO-1/β-actin (**i**) (*n* = 5 per experimental group). **j** The mRNA level of TNF-α was determined by qPCR in the BECs of siRNA-Ctl and siRNA-KLF4-2 group at 12 h restoration of OGD or NO-OGD/R (*n* = 4 per experimental group). NO-OGD/R siRNA-Ctl-treated cells served as control. Data represent mean ± standard deviation and were analyzed by two-way ANOVA. Note that the expressions of three cell adhesion molecules including E-selectin, VCAM-1, and ICAM-1 and the phosphorylation of NF-κB from the siRNA-Ctl-treated bEnd3 cells were significantly induced in response to OGD/R, but the expressions of tight junction proteins (Claudin-5 and ZO-1) markedly decreased compared to that of the controls. Furthermore, OGD/R-induced expression of three cell adhesion molecules and the phosphorylation of NF-κB were enhanced by diminishing the levels of KLF4 in the bEnd3 cells; likewise, the decreased expression of tight junction proteins caused by OGD/R were further augmented by silencing the levels of KLF4 in the bEnd3 cells. OGD/R significantly increased the mRNA expression of TNF-α in the siRNA-Ctl-treated bEnd3 cells, but siRNA-mediated knockdown of KLF4 showed no notable effects on the expression of TNF-α following OGD/R. *P < 0.05, **P < 0.01, ***P < 0.001; ns, not significant

To directly investigate the role and mechanism of KLF4 in regulating the cerebral vascular endothelial inflammation following CIS, we next employed KLF4-specific siRNA to knockdown the expression of KLF4 in bEnd3 cells and performed western blotting to examine the impact of this on the expression of E-selectin, VCAM-1, ICAM-1, and phosphorylation of NF-κB in response to OGD/R. As shown in Fig. 6 a, three different specific siRNA against the murine KLF4 all reduced the expression of KLF4 in bEnd3 cells compared to that of siRNA-Ctl-treated bEnd3 cells, respectively, and siRNA-KLF4-2 can target the KLF4 with the highest efficiency. So, we chose siRNA-KLF4-2 to diminish the expression of KLF4 for later experiments. Twelve hours after restoration, the expressions of three CAMs including E-selectin, VCAM-1, and ICAM-1 and the phosphorylation of NF-κB from the negative control siRNA (siRNA-Ctl)-treated bEnd3 cells were significantly induced relative to the NO-OGD/R siRNA-Ctl-treated controls (*P* < 0.01 for E-selectin, *P* < 0.001 for VCAM-1, *P* < 0.001 for ICAM-1, *P* < 0.001 for phosphorylation of NF-κB). Furthermore, OGD/R-induced expression of three CAMs and the phosphorylation of NF-κB were enhanced by diminishing the levels of KLF4 in the BECs (OGD/R siRNA-KLF4 vs OGD/R siRNA-Ctl: all *P* < 0.01 for E-selectin, VCAM-1, ICAM-1, and phosphorylation of NF-κB) (Fig. 6b–g). These data suggest that KLF4 regulates the expression of CAMs and phosphorylation of NF-κB in bEnd3 cells following OGD/R.

Induction of CMAs including E-selectin and VCAM-1 has been shown to be mediated by the TNF-α-NF-κB pathway in endothelial cells [24]. We wondered if KLF4 suppresses NF-κB phosphorylation through the suppression of TNF-α in OGD-conditioned bEnd3 cells. To examine this, we performed qPCR to determine the mRNA level of TNF-α in the bEnd3 cells of siRNA-Ctl and siRNA-KLF4-2 group at 12 h restoration of OGD or NO-OGD/R. However, as shown in Fig. 6 j, knockdown of KLF4 in bEnd3 cells did not significantly change the mRNA expression of TNF-α after OGD/R.

**Fig. 7:**
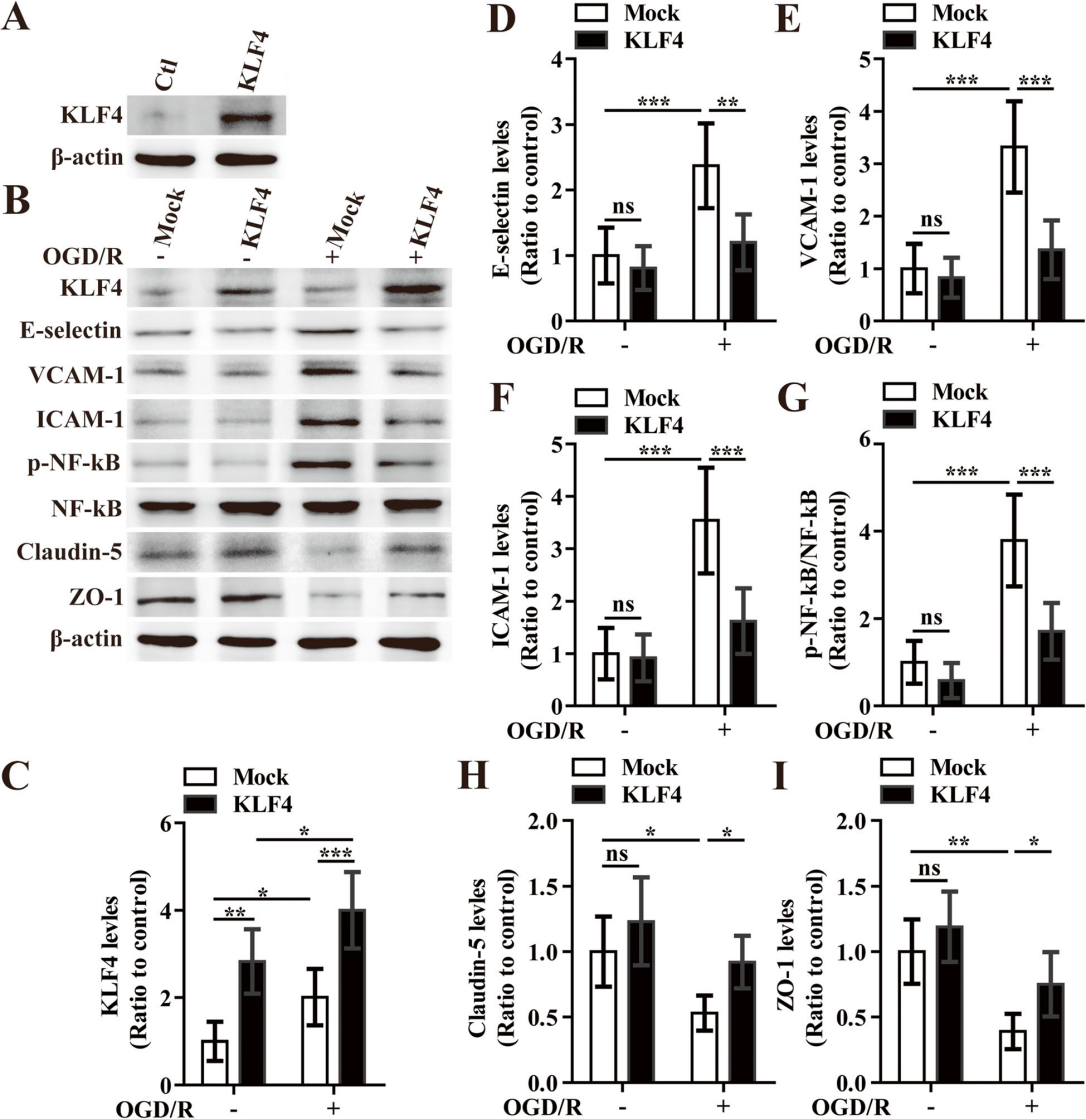
The effect of overexpression of KLF4 on the expression of three cell adhesion molecules, phosphorylated NF-κB, and tight junction proteins in bEnd3 cells under OGD/R conditions. **a** Representative images of western blot for the expression of KLF4 in the bEnd3 cells in the control plasmid (mock) or KLF4 overexpression group. **b** Representative images of western blot for the expression of KLF4, E-selectin, VCAM-1, ICAM-1, p-NF-κB, Claudin-5, and ZO-1 in the bEnd3 cells of mock and KLF4 overexpression group at 12 h restoration of OGD or NO-OGD/R. **c**–**i** Densitometric analysis shows the ratios of KLF4/β-actin (**c**), E-selectin/β-actin (**d**), VCAM-1/β-actin (**e**), ICAM-1/β-actin (**f**), phosphorylated NF-κB/total NF-κB (**g**), Claudin-5/β-actin (**h**), and ZO-1/β-actin (**i**). NO-OGD/R mock-treated cells served as control. Data represent mean ± standard deviation and were analyzed by two-way ANOVA (*n* = 5 per experimental group). Note that the expressions of three cell adhesion molecules including E-selectin, VCAM-1, and ICAM-1 and the phosphorylation of NF-κB on mock-treated bEnd3 cells were significantly induced in response to OGD/R, but the expression of Claudin-5 and ZO-1 markedly decreased compared to that of the NO-OGD/R mock-treated controls. However, these effects were significantly rescued by overexpressing the levels of KLF4 in the bEnd3 cells. *P < 0.05, **P < 0.01, ***P < 0.001; ns, not significant

We then examined how overexpression of KLF4 altered endothelial expression of E-selectin, VCAM-1, ICAM-1, and phosphorylation of NF-κB in response to OGD/R. Consistent with the observations in knockdown experiments, the western analysis revealed that 12 h after restoration, overexpression of KLF4 significantly inhibited the OGD/R-induced expression of three CAMs and the phosphorylation of NF-κB relative the mock-treated group (overexpression of KLF4 vs mock: *P* < 0.01 for E-selectin; *P* < 0.001 for VCAM-1; *P* < 0.001 for ICAM-1; *P* < 0.001 for phosphorylation of NF-κB) (Fig. 7a–g).

KLF4 is supposed to be crucial for the integrity of the blood-tumor barrier because it enhances the promoter activities of tight junction proteins (TJPs), including ZO-1, occludin, and Claudin5 in the glioma endothelial cells [15]. Thus, in the current study, we first performed dual-IF to investigate the relationship between KLF4 and Claudin5 following focal cerebral ischemia. As expected, KLF4 expression co-localized with Claudin-5 on the cerebral blood vessels in the ischemic hemisphere at days 2 and 7 post-ischemia (Fig. 5).

To directly evaluate the role of KLF4 in mediating cerebral vascular permeability following cerebral ischemic stroke, BECs were transfected with the siRNA-Ctl or KLF4-specific siRNA (siRNA-KLF4) for 48 h, then subjected to either 4 h of OGD followed by 12 h of restoration or NO-OGD/R. As shown in Fig. 6 b, h, and i, endothelial expressions of tight junction proteins (TJPs) including claudin-5 and ZO-1 markedly decreased in response to OGD/R as compared to that of the NO-OGD/R siRNA-Ctl-treated controls (*P* < 0.01 for claudin-5; *P* < 0.001 for ZO-1). Moreover, the decreased expression of TJPs caused by OGD/R was further augmented by silencing the levels of KLF4 in the bEnd3 cells (OGD/R siRNA-KLF4 vs OGD/R siRNA-Ctl: *P* < 0.05 for both Claudin-5 and ZO-1). However, in the overexpression experiments, transfection with KLF4 significantly increased Claudin-5 and ZO-1 expression in bEnd3 cells relative to the mock-transfected group under OGD/R conditions (overexpression of KLF4 vs mock: *P* < 0.05 for both Claudin-5 and ZO-1) (Fig. 7b, h, and i).

## Discussion

In the current study, our main findings were as follows: (i) patients with moderate to severe stroke had higher serum levels of three CAMs but lower levels of KLF4 at 48 h after an acute event as compared to patients with minor stroke, and their expression levels correlated well with the infarct volume in all the CIS subjects at that time; (ii) the expressions of three CAMs and KLF4 were all induced in the ischemic hemisphere following focal cerebral ischemia, but the distribution patterns of their expressions were different, where the high levels of KLF4 were expressed, relatively low levels of CAMs were expressed on the cerebral blood vessels, and vice versa; (iii) KLF4 modulated the expressions of CAMs, NF-κB, and TJPs on bEnd3 cells after OGD/R. Taken together, we gave a clearer picture of inter-relationship between endothelial expression of CAMs and KLF4 and BBB dysfunction.

### Serum levels of cell adhesion molecules and KLF4 mirror the severity of ischemic stroke

Inflammatory processes have been shown to be involved in the process of atherogenesis as well as in the pathogenesis of cerebrovascular diseases [2]. CAMs play a crucial role to initiate inflammatory mechanisms soon after cerebral damage [25]. The activated NF-κB and increased CAMs such as ICAM-1 are essential factors involved in ischemia-induced BBB damage [26]. They promote migration of immune cells across the BBB within the cerebral parenchyma, which further exacerbates the brain tissue damage and leads to brain swelling and expansion of the infarction from the penumbra [5]. Moreover, BBB breakdown is reported to be associated with poor prognosis in ischemic stroke [27]. For this reason, the effect of CAMs on the prognosis of ischemic stroke is of high interest. The upregulation of circulating inflammatory adhesion molecules in the peripheral blood has been demonstrated in patients with ischemic stroke within 12 to 72 h after stroke onset, although evidence is contradictory [2]. Interestingly, there was a statistically significant decrease in all CAMs (E-selectin, ICAM-1, and VCAM-1) measured in the peripheral blood in patients who improved clinically on the 4th day compared with the levels on admission, but patients who did not improve had more severe cerebral infarcts, a higher NIHSS score on admission, and no change was observed in levels of CAMs during the short follow-up period [28], suggesting the reduction in CAMs within the first few days of hospitalization may predict a favorable outcome in patients with acute cerebral events. Consistent with these reports, in the current study, we demonstrated that patients with moderate to severe stroke had higher serum levels of all three CAMs at 48 h after an acute event as compared to patients with minor stroke.

There is ample evidence from animal models of middle cerebral artery occlusion that expression of CAMs is associated with cerebral infarct size [2]. In light of this result, we wondered if circulating CAM concentrations are also related to the infarct volume after CIS in a similar manner. In the current study, the Pearson analyses showed that the serum levels of three CAMs (E-selectin, VCAM-1, and ICAM-1) were all positively correlated with infarct volume at 48 h after stroke onset in CIS patients. These results indicate that the CAMs exert a harmful effect on the pathogenesis of acute CIS and serum levels of CAMs reflect the severity of CIS.

As previously reported, treatment of 10% hypertonic saline (HS) not only significantly reduced infarct size induced by MCAO and ipsilateral ischemic hemispheric brain water content, but also increased neurotrophic factors such as interleukin (IL)-10 and IL-4, microglia M2 markers (Arg1, CD206), and KLF4. However, knockdown of KLF4 abrogated the benefits of HS [29]. These findings are in line with our results that serum levels of KLF4 in patients with moderate to severe stroke at 48 h after an acute event were significantly lower than that of the patients with minor stroke. Furthermore, the serum level of KLF4 was negatively correlated with infarct volume at 48 h after stroke onset in all the CIS subjects. These observations suggest that KLF4 may exert a beneficial effect on the pathogenesis of acute CIS and circulating KLF4 might be used as a potential biomarker for predicting the prognosis of acute CIS.

### High expression of KLF4 is always associated with relatively less vascular endothelial inflammation response in the ischemic hemisphere

It is well known that KLF4 suppresses the activation of inflammatory signaling. Its overexpression in endothelial cells induces the expression of multiple anti-inflammatory and anti-thrombotic factors including endothelial nitric-oxide synthase and thrombomodulin, whereas its knockdown enhances TNF-α-induced VCAM-1 expression [10]. Recent evidence has shown that endothelial KLF4 is renoprotective and mediates statin-induced protection against ischemic acute kidney injury (AKI) by regulating the expression of CAMs and concomitant recruitment of inflammatory cells [30]. These results indicate that a tight correlation exists between KLF4 and the CAMs in mediating the inflammatory response in ischemic-related disease*.*

In the current study, we found that in response to cerebral ischemia, the expressions of three CAMs as well as KLF4 were all induced in the ischemic hemisphere following focal cerebral ischemia, but their temporal and spatial expression patterns were different. The expressions of three CAMs including E-selectin, VCAM-1, and ICAM-1 increased in both ischemic penumbra and core, reaching a peak at day 2, and then drastically declining at day 4. While in the ischemic penumbra, the expression of KLF4 increased slightly during the first 2 days post-ischemia, then increased significantly by day 4, and reached a maximum between 7 and 14 days post-ischemia. Obviously, the induction of KLF4 lags behind that of the CAMs in the ischemic penumbra. Though in the ischemic core, the expression of KLF4 changed in a manner similar to that of three CAMs over the 14 days following MCAO.

Interestingly, the dual IF staining showed that where the high levels of KLF4 were expressed, relatively low levels of CAMs were expressed on the cerebral blood vessels in the ischemic hemisphere during the early stage (at day 2) of ischemic stroke, and vice versa. As KLF4 is a key factor in regulating inflammation [10], it seems likely that the enhanced KLF4 suppresses the cerebral vascular endothelial expression of three CAMs after cerebral ischemia.

### KLF4 alleviates cerebral ischemia-induced vascular injury by modulating endothelial expressions of CAMs, NF-κB, and tight junction proteins

A previous study showed that the deletion of KLF4 in endothelial and hematopoietic cells enhanced neointimal formation following vascular injury. The mechanistic analyses revealed that KLF4 inhibited TNF-α-induced expression of VCAM-1 through blocking the binding of NF-κB to the VCAM-1 promoter [11]. Considering these results, we wonder whether this mechanism also occurs after ischemic stroke. To confirm this, we investigated the role of KLF4 in regulating the vascular endothelial inflammation following CIS. Our gene silencing experiment confirmed that diminishing BEC KLF4 expression via selective siRNA-KLF4 resulted in the augmentation of OGD/R-induced expression of three CAMs and phosphorylation of NF-kB. This was further confirmed by the overexpression experiment that transfection of KLF4 significantly inhibited the OGD/R-induced expression of three CAMs and the phosphorylation of NF-κB. These results suggest that KLF4 suppresses cerebral ischemia-induced cerebral vascular inflammation by regulating the expression of CAMs and phosphorylation of NF-κB. Induction of CMAs including E-selectin and VCAM-1 has previously been shown to be mediated by the TNF-α-NF-κB pathway in endothelial cells [24]; thus, KLF4 may suppress NF-κB phosphorylation through suppression of TNF-α in OGD-conditioned BECs. As expected, in the current study, OGD/R significantly increased the mRNA expression of TNF-α in the siRNA-Ctl-treated BECs; however, siRNA-mediated knockdown of KLF4 in BECs showed no notable effects on the expression of TNF-α after OGD/R. It seems that KLF4 does not directly influence the expression of TNF-α but acts to inhibit TNF-α-induced activation of NF-κB to alleviate the cerebral ischemia-induced cerebral vascular inflammation.

Endothelial-specific deletion of both KLF2 and KLF4 was reported to lead to vascular leak in the brain, lungs, kidneys, and heart in the adult animal, suggesting KLF4 is required for maintenance of endothelial and vascular integrity [14]. In addition, KLF4 is shown to regulate the integrity of blood-tumor barrier via enhancing the promoter activities of TJPs including ZO-1, occludin, and Claudin5 in the glioma endothelial cells [15]. Consistent with these results, in the current study, dual-IF staining showed that KLF4 expression co-localized with Claudin-5 on the blood vessels in the ischemic hemisphere at days 2 and 7 post-ischemia, indicating that KLF4 constitutively associated with Claudin5 following focal cerebral ischemia. We further found that endothelial expressions of TJPs including claudin-5 and ZO-1 markedly decreased in response to OGD/R. Of interest, the decreased expression of TJPs caused by OGD/R was further enhanced by knockdown of KLF4 expression in BECs, whereas it was significantly rescued by overexpression of KLF4. This evidence suggests that KLF4 counteracts cerebral ischemia-induced permeability by increasing TJP expression on the blood vessels.

## Conclusions

Our results demonstrate KLF4 conferred vascular protection against cerebral ischemic injury. We provide evidence that serum levels of CAMs and KLF4 mirror the severity of ischemic stroke. And our results suggest that although the expressions of three CAMs and KLF4 are all induced in the ischemic hemisphere following focal cerebral ischemia, the peak timing and distribution patterns of their expression were different: high expression of KLF4 is associate with relatively less vascular endothelial inflammation response in the ischemic hemisphere, and vice versa. Mechanistic analyses reveal that KLF4 alleviates cerebral ischemia-induced vascular injury by modulating endothelial expressions of CAMs, NF-κB, and TJPs. These findings indicate that circulating CAMs and KLF4 might be used as potential biomarkers for predicting the prognosis of acute CIS and also provide a proof of concept for the further translational validation of overexpress of KLF4 for the treatment of CIS patients in clinical practice in the future. In the next set of experiments, we will directly test whether KLF4 alleviates cerebral vascular injury after ischemic stroke by using mice lacking or overexpressing KLF4 in endothelial cells.

## Data Availability

The datasets and materials supporting the conclusions of this article are included within the article.

## Abbreviations

bFGF: Basic fibroblast growth factor
CAMs: Cell adhesion molecules
CIS: Cerebral ischemic stroke
DWI: Diffusion-weighted MRI
ELISA: Enzyme-linked immunosorbent assay
ICAM-1: Inter-cellular adhesion molecule 1
IF: Immunofluorescent
KLF 4: Kruppel-like transcription factor 4
MCAO: Right middle cerebral artery occlusion
MRI: Magnetic resonance imaging
NF-κB: Nuclear factor-κB
NIHSS: National Institutes of Health Stroke Scale
siRNA: Small interfering RNA
TIA: Transient ischemic attack
TNF-α: Tumor necrosis factor
VCAM-1: Vascular cell adhesion molecule 1
